# Energy-Efficient Single Layer Spin Hall Nano-Oscillators Driven by Berry Curvature

**DOI:** 10.1021/acsnano.5c02048

**Published:** 2025-05-09

**Authors:** Lakhan Bainsla, Yuya Sakuraba, Akash Kumar, Avinash Kumar Chaurasiya, Keisuke Masuda, Nattamon Suwannaharn, Ahmad A. Awad, Nilamani Behera, Roman Khymyn, Taisuke Sasaki, Saroj Prasad Dash, Johan Åkerman

**Affiliations:** † Department of Physics, Indian Institute of Technology—Ropar, Roopnagar, Punjab 140001, India; ‡ Department of Physics, 3570 University of Gothenburg, Göteborg 41296, Sweden; § Department of Microtechnology and Nanoscience, Chalmers University of Technology, Göteborg 41296, Sweden; ∥ Research Center for Magnetic and Spintronic Materials, 52747 National Institute for Materials Science, 1-2-1, Sengen, Tsukuba, Ibaraki 305-0047, Japan; ⊥ Center for Science and Innovation in Spintronics, Tohoku University, 2-1-1 Katahira, Aoba-ku, Sendai 980-8577, Japan; # Research Institute of Electrical Communication, Tohoku University, 2-1-1 Katahira, Aoba-ku, Sendai 980-8577, Japan; ¶ Wallenberg Initiative Materials Science for Sustainability, Department of Microtechnology and Nanoscience, Chalmers University of Technology, Göteborg 41296, Sweden; ∇ Graphene Center, Chalmers University of Technology, Göteborg 41296, Sweden

**Keywords:** magnetic Weyl semimetal, Berry curvature, intrinsic spin−orbit torque, spin Hall nano-oscillator, magnetization auto-oscillations, microfocused Brillouin light scattering

## Abstract

Spin Hall nano-oscillators (SHNOs) are emerging spintronic oscillators with significant potential for technological applications, including microwave signal generation, and unconventional computing. Despite their promising applications, SHNOs face various challenges, such as high energy consumption and difficulties in growing high-quality thin film heterostructures with clean interfaces. Here, single-layer topological magnetic Weyl semimetals open a possible solution as they possess both intrinsic ferromagnetism and a large spin–orbit coupling due to their topological properties. However, producing such high-quality thin films of magnetic Weyl semimetals that retain their topological properties and Berry curvature remains a challenge. We address these issues with high-quality single-layer epitaxial ferromagnetic Co_2_MnGa Weyl semimetal thin film-based SHNOs. We observe a giant spin Hall conductivity, σ_SHC_ = (6.08 ± 0.02) × 10^5^ (ℏ/2*e*) Ω^–1^ m^–1^, which is an order of magnitude higher than previous reports. Theoretical calculations corroborate the experimental results with a large intrinsic spin Hall conductivity due to presence of a strong Berry curvature. Further, self spin-orbit torque driven magnetization auto-oscillations are demonstrated for the first time, at an ultralow threshold current density of *J*_th_ = 6.2 × 10^11^ A m^–2^. These findings indicate that magnetic Weyl semimetals have tremendous application potential for developing energy-efficient spintronic devices.

## Graphical abstract

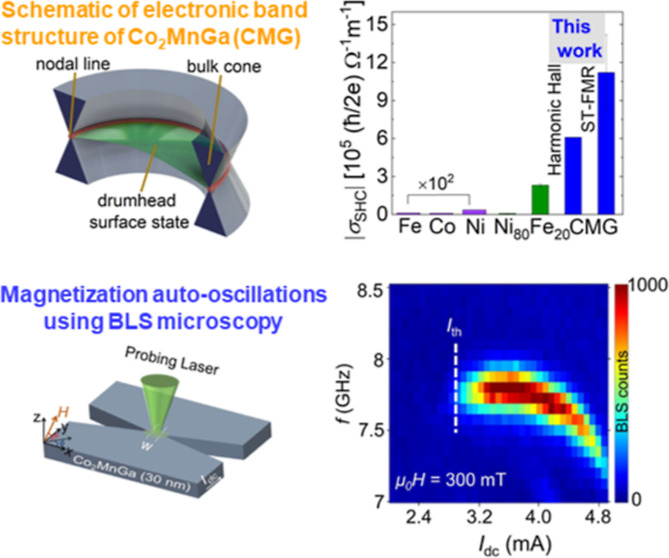

## Introduction

Spin Hall nano-oscillators (SHNOs) are emerging spintronic devices that leverage the spin Hall effect in heavy metals (HM, e.g. Pt, W, Ta) to convert charge currents into spin currents and use the resulting spin-orbit torque (SOT) to drive high-frequency magnetization precession in an adjacent ferromagnetic (FM) layer.^1,2^ Thanks to their easy fabrication, large microwave frequency tunability with both magnetic field and current, and propensity for mutual synchronization in large 2D arrays, SHNOs show great promise for applications in microwave signal generation,^3^ Ising machines,^4^ and unconventional computing.^5,6^ While straightforward to both deposit and pattern, the HM/FM bilayer structure of SHNOs has a number of disadvantages: (i) the current through the FM does not generate any SOT, (ii) the current through the HM does not generate any microwave signal,^3^ and (iii) substantial spin current is lost at the HM/FM interface due to spin memory loss.^7^ As a remedy, single-layer SHNOs were recently demonstrated using ultranarrow SHNOs made from conventional NiFe films with broken inversion symmetry from different oxide interfaces.^8^ However, the resulting charge-to-spin conversion was low and, consequently, the resulting current densities of 2.7 × 10^12^ A m^–2^ were very high. To dramatically improve on the intrinsic charge-to-spin conversion, one may look to topological Weyl semimetals (WSMs), which are an emerging class of materials, where the nontrivial topological properties arising from Weyl Fermions in the bulk nodes as well as Fermi arcs surface states offer both enhanced spin current generation and reduced energy dissipation.^9^ Additionally, these quantum materials show a large electric-field effect on their properties which is further helpful in developing energy-efficient spintronic devices.^9–11^ These materials are already showing potential in various spintronic applications, such as spin-polarized current generation, magnetoresistive devices, spin-based logic operations, and THz generation.^9,12–16^ Magnetic WSMs that break time-reversal symmetry are particularly attractive as they allow interplay between magnetism and topology in a single material and are expected to result in a large intrinsic anomalous Hall and spin Hall conductivity due to the large intrinsic Berry curvature.^9,17–19^ Such magnetic WSMs can be highly useful to obtain spintronic devices based on single magnetic layer overcoming the above-mentioned challenges. Recently, several ferromagnetic WSMs have been discovered confirming their Weyl nodes and Fermi arc surface states.^17–20^ Interestingly, topological Weyl Fermionic bands and ferromagnetism up to a *T*_C_ of 690 K have been discovered in Heusler alloy Co_2_MnGa (CMG).^17,18,21^ Very recently, SOT has been reported in *B*2 and less *L*2_1_-ordered CMG thin films^22–24^ and it has been found that the efficiency of SOT components is comparable to trivial systems. It is hence important to obtain high-quality *L*2_1_-ordered CMG thin films to realize the full potential of Berry curvature-induced giant SOT for spintronic applications.^25,26^

Here, we first develop the most highly ordered single-layer epitaxial CMG thin films to date and then demonstrate giant self-induced SOTs and energy-efficient single-layer nano-oscillators at room temperature, driven by their pronounced topological properties and strong Berry curvature. We investigate epitaxial ferromagnetic CMG thin films of 10–30 nm with high structural order, showing very high values of anomalous Hall conductivity, σ_*xy*_ = 1.35 × 10^5^ Ω^–1^ m^–1^, and an anomalous Hall angle, θ_H_ = 15.8%, both highest so far in the thin films and comparable to bulk crystal values^17,18,27,28^ confirming their topological order and Berry curvature. Using harmonic Hall measurements, we observe a giant intrinsic spin Hall conductivity, σ_SHC_ = (6.08 ± 0.02) × 10^5^ (ℏ/2*e*) Ω^–1^ m^–1^, which is an order of magnitude higher than literature values of bilayer CMG/CoFeB^22–24^ and single-layer ferromagnets.^29,29,30^ The obtained spin Hall conductivity results are further validated by the spin-torque ferromagnetic resonance (ST-FMR) technique. Consequently, self-SOT-driven magnetization auto-oscillations are observed in a single-layer magnetic WSM nanoconstriction SHNOs using microfocused Brillouin light scattering (μ-BLS) microscopy,^31,32^ at an ultralow threshold current density of *J*_th_ = 6.2 × 10^11^ A m^–2^, a factor of 5 lower than that previously reported for NiFe.^8^ Theoretical calculations of the intrinsic spin Hall conductivity, originating from a strong Berry curvature, corroborate the results and yield values comparable to the experiment.

## Results and Discussion

### Structural and Magnetic Characterization

The quality of thin films is critical for achieving the desired properties in magnetic Weyl semimetals. To assess the growth quality, the out-of-plane 2θ–θ X-ray diffraction (XRD) measurements were performed on CMG films with different thicknesses, as shown in Figure 1a. The (002) superlattice peaks and (004) fundamental peaks are clearly visible in the XRD patterns for all the samples, indicating that the films were grown with (001) crystalline orientation and high *B*2 structural ordering (*B*2 refers to the ordering between Co and Mn-Ga). Further, the epitaxial growth of the films was confirmed by performing ϕ scans for the CMG (220) fundamental peak. A clear 4-fold rotation symmetry confirms the epitaxial growth for all films (see Supporting Information, Figure S1a). The (111) superlattice peak of CMG was observed for the 20 and 30 nm films, confirming *L*2_1_ structural order (*L*2_1_ refers to the ordering between Mn and Ga). The (111) peak is absent in the 10 nm film, which may be due to lower *L*2_1_-ordering in thinner films or its intensity being below the detection limit of our XRD system (see Supporting Information, Figure S1b). The *B*2 and *L*2_1_ structural ordering parameters *S*_*B*2_ and *S*_L2_1__ were estimated using the experimental and calculated XRD intensity ratios,^33^ and the estimated values are plotted in Figure 1b. The 30 nm film shows a slightly higher value of *S*_*L*2_1__ compared to the 20 nm film. The obtained values of *S*_*B*2_ and *S*_*L*2_1__ confirm full *B*2 and partial *L*2_1_ ordering, substantially better than earlier thin film reports on this material.^22–24^

**Figure 1:**
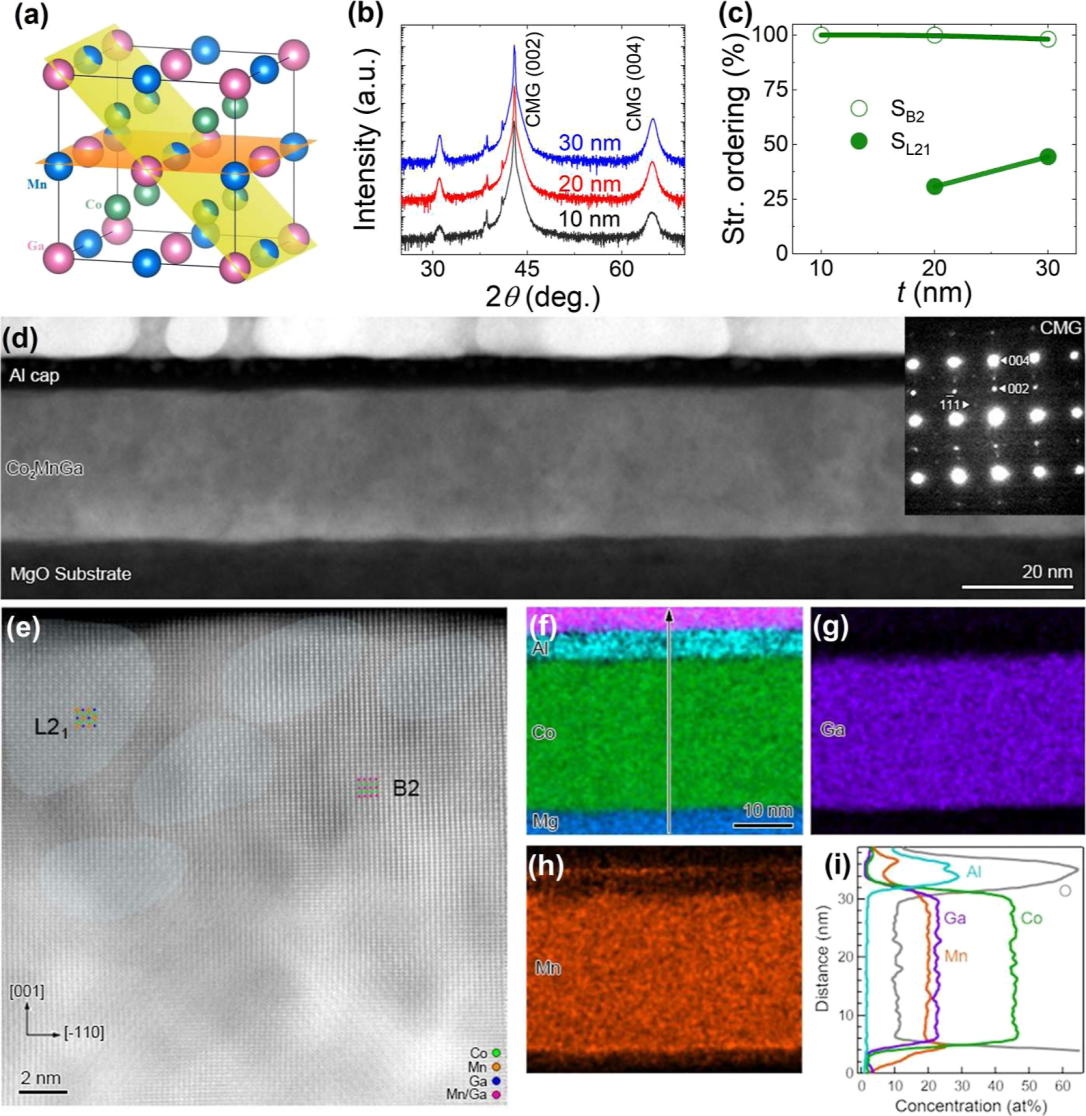
(a) The crystal structure of the *L*2_1_-ordered CMG Heusler alloy. (b) Out-of-plane XRD patterns for CMG films with three different thicknesses. (c) Structural ordering parameters *S*_*B*2_ and *S*_*L*2_1__ vs film thickness; lines are guides to the eye. (d) Low-magnification HAADF–STEM image with the NBED pattern obtained from the zone axis of CMG (110), indicating the (001) oriented growth, in the inset. (e) A high-magnification HAADF–STEM image viewed along the (110) zone axis with the schematic illustrations of *L*2_1_ and *B*2-ordered structure. Note that the regions highlighted with light blue color represent the *L*2_1_-ordered region. EDS elemental maps of (f) Al, Co, Mg, (g) Ga, (h) Mn. (i) Line compositional profile acquired along the direction marked by the arrow in (f).

In the 30 nm thick CMG sample, which exhibited the highest *S*_*L*2_1__, the cross-sectional high-angle annular dark-field (HAADF) scanning transmission electron microscopy (STEM) image confirmed the uniform thickness of both the CMG and Al layers, with relatively smooth interfaces, as shown in Figure 1d. The nanobeam electron diffraction (NBED) pattern of the CMG layer, obtained from the (110) zone axis of CMG, reveals (001) oriented growth of the CMG layer, as indicated in the inset of Figure 1d. The presence of the (111) superlattice reflection further confirms the *L*2_1_ ordered structure, which is consistent with the XRD results. However, the high-magnification HAADF–STEM image viewed along the (110) zone axis of the CMG layer shows that it consists of a mixture of *L*2_1_ and *B*2-ordered regions, as depicted in Figure 1e. In the areas highlighted in light blue, an atomic layer with alternating brightly and darkly imaged atomic columns, along with a layer consisting solely of darkly imaged atomic columns, are stacked alternately along the (002) direction. In contrast, the unhighlighted regions exhibit alternating stacking of layers with both brightly and darkly imaged atomic columns. Given that the Ga and Mn columns in the *L*2_1_-ordered CMG layer can be distinguished from the (110) zone axis (as illustrated in the schematic of Figure 1e), and that the HAADF–STEM image intensity is proportional to the square of the atomic number (*Z*), it can be inferred that the light blue-highlighted areas correspond to *L*2_1_-ordered regions, while the unhighlighted areas correspond to *B*2-ordered regions. Figure 1f–i present energy-dispersive X-ray spectroscopy (EDS) elemental maps of the constituent elements, along with line compositional profiles analyzed along the direction indicated in Figure 1f. Within the CMG layer, Co, Mn, and Ga are uniformly distributed, indicating the absence of secondary phases. The EDS line compositional profile further confirms the uniformity of the chemical composition within the CMG layer, which is estimated to be Co_51.8_Mn_22.6_Ga_25.6_.

To probe the presence of Weyl features (as shown in Figure 2a) and Berry curvature reported in earlier studies,^17,18,20^ Hall conductivity measurements were performed in the temperature range of 50–300 K. Anomalous Hall conductivity values of about 0.45 × 10^5^ and 1.35 × 10^5^ Ω^–1^ m^–1^ were obtained at a temperature of 50 K for the 20 and 30 nm CMG films, as shown Figure 2b,c, respectively. These values are higher than those reported for thick (∼80 nm; 1138 S/cm at 2 K) CMG films^27^ and comparable to the bulk case.^17^ The origin of the large anomalous Hall conductivity was discussed in an earlier work by comparing measured angle-resolved photoemission spectroscopy data to the Berry curvature obtained from density functional theory (DFT) calculations and was attributed to the presence of a large Berry curvature associated with Weyl lines in the CMG electronic band structure.^17,20^ Our high value of anomalous Hall conductivity for the 30 nm film suggests the presence of Weyl lines and corresponding Berry curvature. The anomalous Hall angle, θ_H_ = σ_*xy*_^AHE^/σ_*xx*_, which reflects the ability of a material to deviate the electron flow from the direction of the longitudinal electric field, was estimated using transport measurements (see Supporting Information, Figure S4 for electrical conductivity, σ_*xx*_, measurements), and a record high-value of θ_H_ = 15.8% was obtained for the 30 nm film.

**Figure 2:**
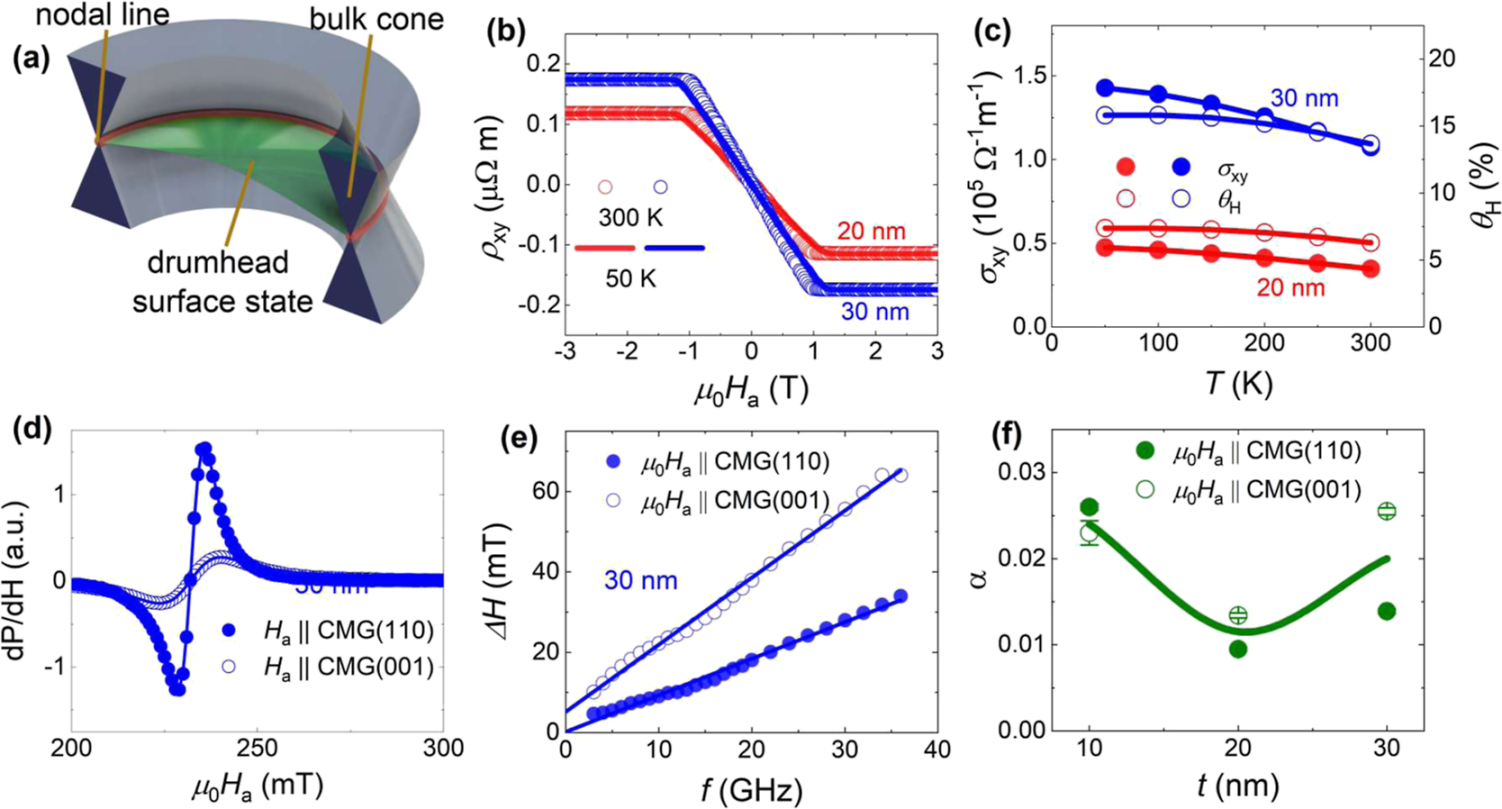
(a) Schematic of the band structure of CMG showing the bulk cone, nodal line (orange) and drumhead surface states (green). (b) Hall resistivity, ρ_*xy*_, vs applied magnetic field, μ_0_ *H*_a_, at 50 and 300 K for the 20 and 30 nm films. (c) Temperature-dependent anomalous Hall conductivity, σ_*xy*_, and anomalous Hall angle, θ_H_, for the 20 and 30 nm films; lines are guides to the eye. (d) Ferromagnetic resonance spectra for 30 nm film. (e) Ferromagnetic resonance linewidth, Δ*H*, vs *f* for CMG 30 nm film. (f) Gilbert damping constant as a function of CMG thickness. (d–f) Solid and open symbols represent the experimental data points when applied magnetic field, μ_0_ *H*_a_, is parallel to CMG (110) and (001) planes, respectively. (d,e) Solid lines are fit to the experimental data,^34^ while solid lines in (f), are just guide to eye.

The Gilbert damping constant α is an important parameter in optimizing energy-efficient spintronic devices such as SHNOs^5,35–37^ and to obtain its values, the room temperature broadband ferromagnetic resonance (FMR) measurements were performed in the frequency range *f* = 3–38 GHz with the magnetic field, *H*_a_, applied parallel to the CMG (110) and (001) planes, as given in Figure 2d for 30 nm film. The resonance field (*H*_R_) and the linewidth (Δ*H*) were extracted by fitting the experimental data to a sum of symmetric and antisymmetric Lorentzian derivatives.^34^ The effective magnetization, μ_0_ *M*_eff_, was obtained from fits of *f* vs *H*_R_ to Kittel’s equation;^34,38^ μ_0_ *M*_eff_ ranges from 0.75 T to 0.9 T for the three films of different thicknesses (see the Supporting Information, Figure S3). The α was obtained from linear fits of Δ*H* vs *f* to Δ*H* = Δ*H*_0_ + (4πα*f*)/γμ_0_ (Figure 2e); here γ/2π is the effective gyromagnetic ratio and μ_0_ is the permeability of free space. The obtained α values are slightly higher than previously reported values,^39^ but show a very interesting hitherto unexplored crystalline orientation dependence, as α_001_ = 0.025 ± 0.001 (*H*_a_ applied along the (001) plane) is almost twice as large as α_110_ = 0.014 ± 0.001 (*H*_a_ applied along the (110) plane) for the 30 nm film; see Figure 2f. The μ_0_ *M*_eff_ values are in agreement to the obtained saturation magnetization of, 4π*M*_S_ = 0.84 ± 0.02 T, for the 30 nm film using vibrating sample magnetometer measurements. The obtained 4π*M*_S_ values (see Supporting Information, Figure S2) are comparable to earlier reports on thin films,^22,23^ but less than the bulk values, 4π*M*_S_ = 0.96–0.98 T.^17,40^

### Giant Self-Induced Spin–Orbit Torques

To quantify the self-induced SOTs harmonic Hall measurements were performed on the 20 and 30 nm CMG films, as shown in the schematic in Figure 3a. The SOT efficiency was estimated using fits of the second harmonic (*R*_2ω_) Hall resistance vs ϕ_H_, to^41,42^

1 R2ω=−(RAHEHADHa−Hkeff+RT)cos⁡ϕH+2RPHEHFL+HOeHa(2cos3ϕH−cos⁡ϕH)

where *R*_AHE_ and *R*_PHE_ are the anomalous and planar Hall resistances, *H*_FL_, *H*_AD_ and *H*_Oe_ are the effective field of the field-like torques, the effective field of the antidamping-like torques and the current induced Oersted field, respectively. *R*_T_ is the second harmonic Hall resistance signal due to the thermo-electric effects including the anomalous Nernst effect and spin Seebeck effect. The measured *R*_2ω_ vs ϕ_H_ was fitted with eq 1 and separated into cos ϕ_H_ and 2cos^3^ϕ_H_ – cos ϕ_H_ contributions. The cos ϕ_H_ contribution of *R*_2ω_ (*R*_2ω_^cosϕ^) vs 1/(μ_0_ *H*_a_ – μ_0_ *H*_k_^eff^) for the 30 nm CMG film is shown in Figure 3c; *H*_AD_ and *R*_T_ values were obtained from the slope and *y*-intercept of the linear fit of the data, respectively. We assume no net Oersted field contribution in our case, as we are working with a single CMG layer. Figure 3d,e show the obtained *H*_AD_ vs *J* (current density) for the 20 and 30 nm films, respectively. The slope of *H*_AD_ vs *J* was obtained from the linear fit and used to evaluate the effective antidamping-like torque efficiency, ξ_AD_^eff^, with the relation^42^

2 $$\xi_{AD}^{eff}=\frac{2em_{S}}{\hbar }\frac{\delta \mu_{0}H_{AD}}{\delta J}$$

where, *e* is the elementary charge, ℏ is the reduced Planck’s constant, and *m*_S_ is the saturation magnetization per unit area. ξ_AD_^eff^ values of 0.24 ± 0.01 and 0.82 ± 0.02 are obtained for the 20 and 30 nm films, respectively (see Supporting Information, Figure S5 for CMG 20 nm data). The obtained value of ξ_AD_^eff^ = 0.82 ± 0.02 is an order of magnitude higher than the previously reported values for CMG/Ti/CoFeB^22,23^ and about 5 times the value reported for single layer (110) oriented CMG films.^24^ The effective spin Hall conductivity, σ_SHC_ = σ_*xx*_ξ_AD_^eff^ = (1.24 ± 0.01) × 10^5^ and (6.08 ± 0.02) × 10^5^ (ℏ/2*e*) Ω^–1^ m^–1^ were estimated for the 20 and 30 nm CMG films, respectively. The σ_SHC_ value for the 30 nm CMG film is at least an order of magnitude higher than reported values for other single layer magnets such as Ni_80_Fe_20_,^8,29,30^ Ni, Fe, and Co.^29^ Moreover, it remains higher—by an order of magnitude—compared to other topological materials such as TaAs,^43^ WTe_2_,^44^ Bi_2_Se_3_,^45^ Bi_2_Te_3_ ^46^ etc.

**Figure 3:**
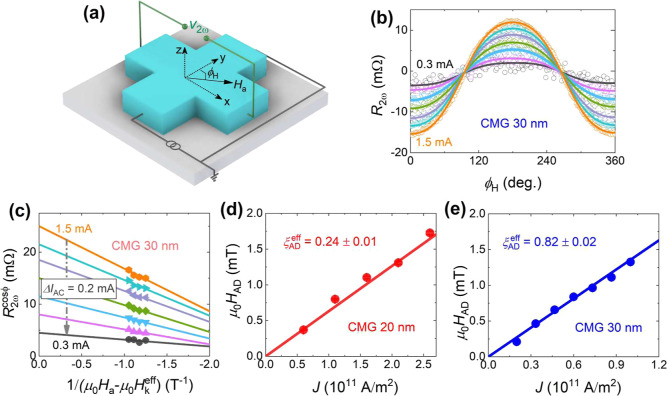
(a) Schematic of the measurement set up showing the directions of alternating current, *I*_AC_, and applied magnetic field, *H*_a_ with coordinates. (b) Second harmonic Hall resistance, *R*_2ω_, of the 30 nm CMG film vs in-plane magnetic field angle, ϕ_H_, in an applied magnetic field μ_0_ *H*_a_ = 0.2 T, for seven different alternating current values *I*_AC_ = 0.3–1.5 mA, in steps of 0.2 mA. Open symbols are the experimental data points; solid lines are fits to eq 1. (c) The cos ϕ contribution to *R*_2ω_ (*R*_2ω_^cosϕ^) as a function of 1/(μ_0_ *H*_a_ – μ_0_ *H*_k_^eff^) at the seven different current values. Filled symbols are the experimental data points; solid lines are linear fits. (d,e) The antidamping-like field μ_0_ *H*_AD_ vs current density *J* for 20, and 30 nm films, respectively. Solid symbols are the experimental data points; solid lines are linear fits. The extracted effective antidamping-like torque value, ξ_AD_^eff^, is shown in the figures.

To further estimate the SOT efficiency, ST-FMR measurements were performed on rectangular 4 × 14 μm^2^ microstrips fabricated with the longer axis along the CMG (110) plane (measurement schematic is given in Figure 4a). We chose the CMG (110) plane because it exhibits the lowest Gilbert damping as discussed above in FMR results and the highest SOT efficiency.^24^ Anisotropic magnetoresistance measurements were performed on the ST-FMR microstrips and negative anisotropic magnetoresistance values in the range from −0.37 T to −0.41 T were obtained, as shown in Supporting Information, Figure S6. A representative ST-FMR signal (*V*_mix_) for the 30 nm film is shown in Figure 4b; we did not observe a clear signal for the 20 and 10 nm films which might be due to the loss of topological features as these films have relatively lower structural ordering. The obtained *V*_mix_ was fitted to a single Lorentzian function, which is the sum of symmetric and antisymmetric components.^15,47,48^ The μ_0_ *M*_eff_, and α values are obtained in the same way as mentioned in the FMR analysis (raw data and fits shown in Supporting Information, Figure S7). The obtained values μ_0_ *M*_eff_ = 0.79 ± 0.04 T, and α = 0.018 ± 0.001 are comparable to the FMR results on the blanket films when *H*_a_ applied along the (110) plane.

**Figure 4:**
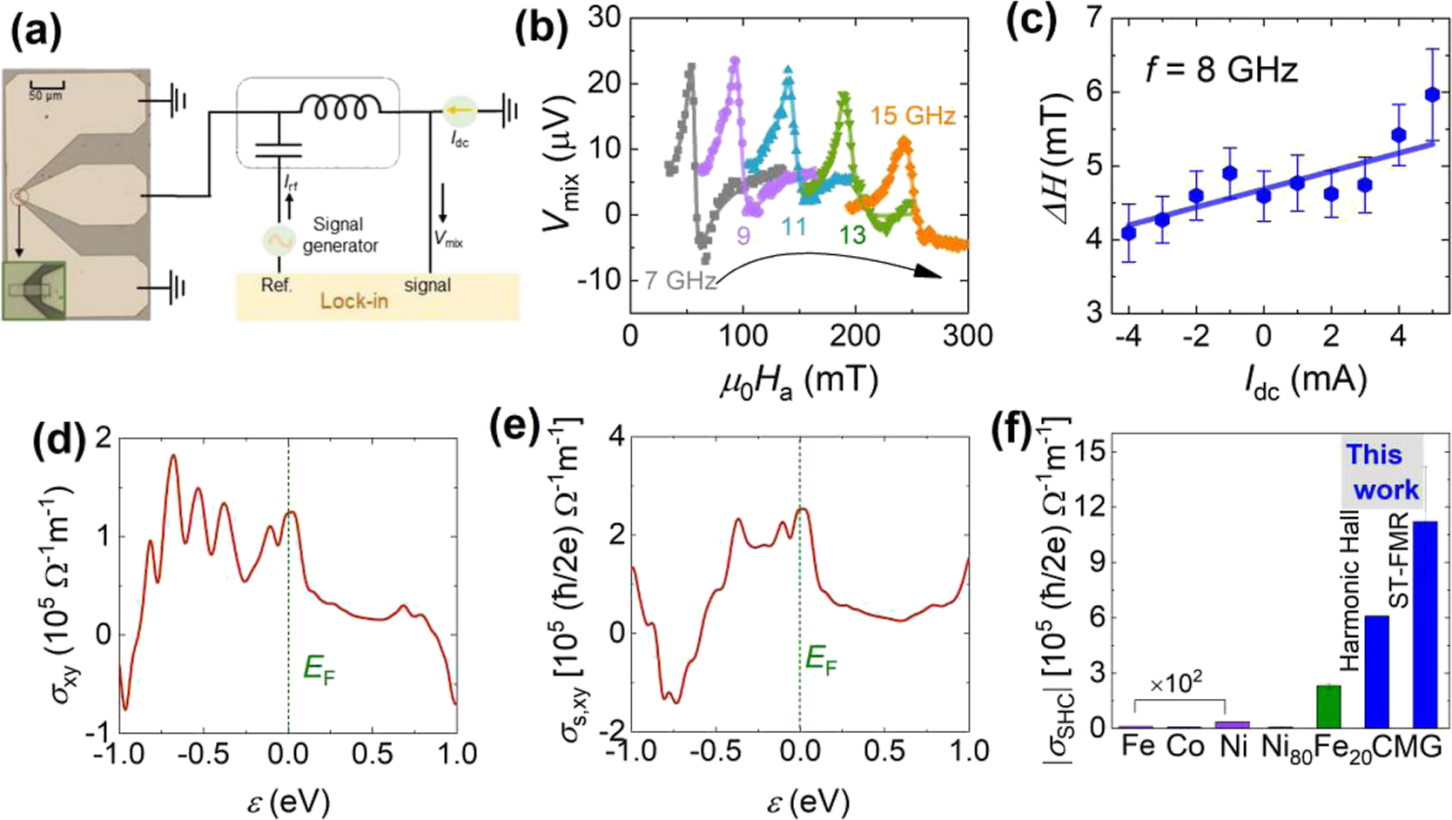
(a) Schematic of the ST-FMR measurement setup. (b) Five representative STFMR curves in the frequency range *f* = 7–15 GHz. Solid symbols are the experimental data; solid lines are fit to, *V*_mix_ = *SF*_S_(*H*_a_) + *AF*_A_(*H*_a_).^47,48^ (c) Resonance linewidth Δ*H* vs dc bias *I*_dc_ measured at a frequency of 8 GHz, here solid symbols are the data points obtained by fitting the *V*_mix_ signal and the solid line is a linear fit to the obtained data. (b,c) All the measurements are done with ϕ_H_ = 60°. (d,e) The calculated anomalous Hall and spin Hall conductivities as a function of ϵ being the energy relative to the Fermi energy. The dashed line in (d,e) refers to the position of the Fermi level (energy), *E*_F_. (f) Comparison of the experimental spin Hall conductivity, σ_SHC_ values obtained for CMG with values reported for other single-layer magnetic materials.^8,29^

The current dependent ST-FMR measurements were carried out at a fixed frequency (8 GHz) to estimate the effective antidamping-like torque efficiency (θ_AD_^eff^). The change in Δ*H* vs applied dc current, *I*_dc_, at a frequency of 8 GHz is shown in Figure 4c. The slope (δΔ*H*/δ*I*_dc_) of linearly fit Δ*H* vs *I*_dc_ from Figure 4c can be used to extract θ_AD_^eff^ ^15,47,48^

3 $$\theta_{AD}^{eff}=\frac{2e}{\hbar }\frac{\left(H_{a}+0.5M_{eff}\right)\mu_{0}M_{s}t_{CMG}}{\sin\phi_{H}}\frac{\gamma }{2\pi f}\frac{\delta \Delta H}{\delta I_{dc}}A_{c}$$

where, *t*_CMG_ is the thickness of the CMG layer, *A*_c_ the cross-sectional area of the ST-FMR microbars, and ϕ_H_ the angle between the applied magnetic field and the rf/dc current. A θ_AD_^eff^ value of 1.58 ± 0.40, and an effective spin Hall conductivity, σ_SHC_ = σ_*xx*_θ_AD_^eff^, value of (1.12 ± 0.30) × 10^6^ (ℏ/2*e*) (Ω m)^−1^ were estimated for the 30 nm CMG film, corroborating the very high value achieved above using the harmonic Hall measurements.

To gain further theoretical insight into the giant spin Hall effect, we calculated the spin Hall conductivity σ_s,*xy*_ in *L*2_1_-ordered CMG by combining first-principles calculation with the Kubo formula. The spin Hall conductivity σ_s,*xy*_ can be calculated in the same way as the anomalous Hall conductivity σ_*xy*_ except that the usual momentum operator *p*_*y*_ is replaced by *p*_*y*_^s^ = {*p*_*y*_,*s*_*z*_}. Figure 4d shows the energy dependence of the anomalous Hall conductivity σ_*xy*_, where ϵ = 0 corresponds to the Fermi level in the present system. We obtained a large value of σ_*xy*_ (∼1.2 × 10^5^ Ω^–1^ m^–1^) at ϵ = 0, consistent with previous studies.^18,20,49^ It is known that *L*2_1_-ordered CMG has several mirror symmetries in its crystal structure, and these provide Weyl nodal loops in the *k*_*i*_ = 0 plane (*i* = *x*, *y*, *z*) in the Brillouin zone, as shown in the schematic of Figure 1d.^17,20,40,49,50^ When the spin-orbit interaction is taken into account, some of these nodal loops are gapped and yield a large value of the Berry curvature, which is the reason for the very large σ_*xy*_.^20,40,49^ In Figure 4e, we show the energy dependence of the spin Hall conductivity σ_s,*xy*_. We obtained a large σ_s,*xy*_ of ∼1.3 × 10^5^(ℏ/*e*) Ω^–1^ m^–1^ at ϵ = 0, which is comparable to the experimentally obtained large values. Since σ_s,*xy*_ is given as the integral of the spin Berry curvature in the Brillouin zone, the gapped nodal loops are also considered to be the origin of the large spin Hall conductivity. These results clearly indicate that the experimentally obtained large spin Hall effect can be understood by the intrinsic mechanism originating from the electronic structure of *L*2_1_-ordered CMG.

### Ultralow Current Density SHNOs

To confirm and use the observed giant intrinsic SOT, we fabricated 150 nm wide nanoconstriction SHNOs^8,36^ out of the 30 nm CMG film and measured their spin wave spectra as a function of field and SHNO current using μ-BLS microscopy. Figure 5a shows the schematic of the μ-BLS measurements with the SHNO layout, the magnetic field geometry, and the laser spot (∼300 nm) focused onto the center of the nanoconstriction. The external magnetic field is applied at an out-of-plane angle, θ = 60° and an in-plane angle, ϕ = 0°. Figure 5b shows a scanning electron microscopy image of the SHNO. Additional details of the SHNO device fabrication and μ-BLS measurements are described in the Methods section.

**Figure 5:**
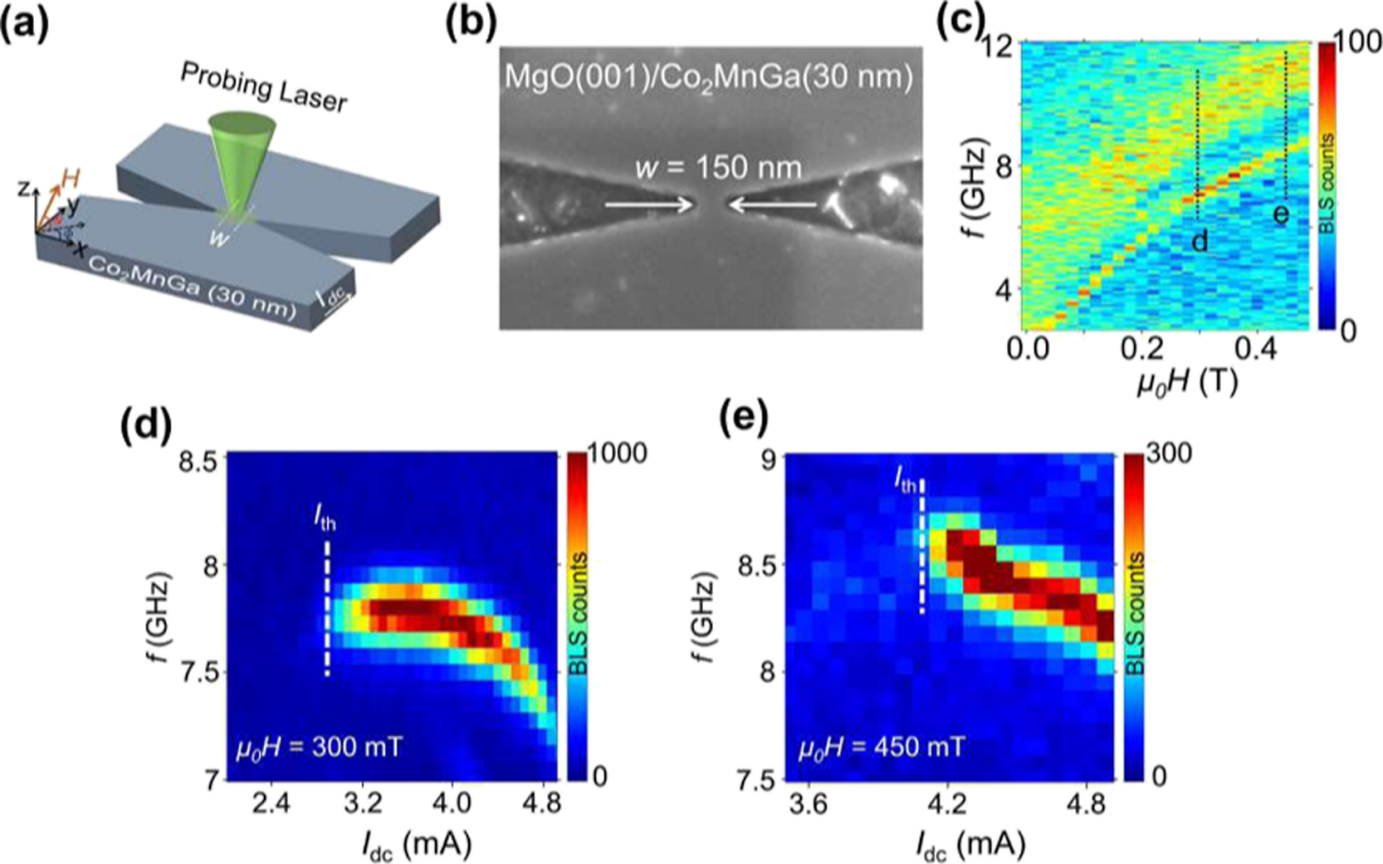
(a) Schematic of the μ-BLS measurement geometry. (b) SEM image of the fabricated 150 nm wide nanoconstriction SHNO based on the 30 nm CMG film. (c) μ-BLS measurements of the thermal spin wave spectral distribution vs field strength measured at *I*_dc_ = 0. (d,e) Current dependent auto-oscillation signal measured in a 60° out-of-plane magnetic field μ_0_ *H* = 300 mT, and 450 mT, respectively. The threshold current (*I*_th_) is marked with dashed white line in each plot.

Figure 5c shows the spectral distribution of the thermal (zero current) BLS counts vs field strength. As expected for a material without perpendicular magnetic anisotropy, we observe a wide band of spin waves with a lower FMR cutoff following a Kittel-like frequency-vs-field dependence and a higher more gradual decay as the spin waves approach the wave vector resolution limit of the BLS microscope. Well below the FMR cutoff, we also observe a narrow spin wave mode, which we identify as the typical nanoconstriction edge mode.

Figure 5d,e show the corresponding spin wave spectral distribution vs SHNO current for two different magnetic fields of 300 mT and 450 mT. The thermal BLS counts are now entirely swamped by spin wave auto-oscillations starting at about 2.8 and 4.1 mA, respectively. The auto-oscillations occur on the nanoconstriction edge mode with a frequency that decreases with current, consistent with the negative nonlinearity.^51^

To confirm the first estimates of the auto-oscillation threshold currents, we fit a Lorentzian to the BLS spectral distribution at each current and plot the integrated BLS counts and the distribution width vs SHNO current as shown in Supporting Information, Figure S8. At threshold, the integrated BLS counts show a sharp change of slope and the extracted spectral linewidth drops to the instrument frequency linewidth of the BLS microscope (∼200 MHz). Using these observations as approximate criteria for the threshold current, we extract 2.8 mA at 300 mT and 4.1 mA at 450 mT. This translates into threshold current densities of *J*_th_ = 6.2 × 10^11^ A m^–2^ and *J*_th_ = 9.1 × 10^11^ A m^–2^, which is 1 order of magnitude lower than that of 15 nm single-layer NiFe nano oscillators.^8^ Considering that CMG and NiFe have about the same saturation magnetization and that the film in our CMG SHNO is twice as thick as the NiFe in,^8^ the ultralow threshold current density is a strong confirmation of the giant intrinsic SOT of CMG.

## Conclusion and Outlook

In this study, we demonstrate very high values of σ_*xy*_ and σ_SHC_ in epitaxial ferromagnetic CMG films and operation of single-layer magnetic Weyl semimetal spin Hall nano-oscillators at ultralow threshold current densities. High, bulk-like, values of the anomalous Hall conductivity, σ_*xy*_ = 1.35 × 10^5^ Ω^–1^ m^–1^, and the anomalous Hall angle, θ_H_ = 15.8% supports the presence of topological surface states and large Berry curvature.^17^ The SOT efficiency, measured using second harmonic Hall resistance and corroborated with ST-FMR, yields a σ_SHC_ value of (6.08 ± 0.02) × 10^5^ (ℏ/2*e*) Ω^–1^ m^–1^ for the 30 nm CMG film, which is an order of magnitude higher than previously reported for single-layer magnets and multilayer Co_2_MnGa stacks. Theoretical calculations further support this observation, attributing the giant intrinsic SOT to large Berry curvature.

The single-layer ferromagnetic SHNOs, using TSMs with large SOTs and ultralow current densities, will offer improved energy efficiency by reducing heat, eliminating shunting and interfacial spin-memory loss, and maximizing output power through effective magnetoresistance utilization.^8,52^ Benefiting from topological surface states and large Berry curvature, we demonstrate single-layer magnetic WSM with a high charge-to-spin conversion and ultralow current density (*J*_th_ = 6.2 × 10^11^ A m^–2^) operation of SHNOs. The operating current and current density of SHNOs can be further reduced by optimizing ultrathin (<10 nm) epitaxial CMG films with high crystal order and lower Gilbert damping while retaining a large θ_AD_^eff^.^34^

Due to their simple nanofabrication process and exceptional mutual synchronization, nanoconstriction SHNOs are rapidly emerging as promising spintronic oscillators for applications in wireless communication and unconventional computing.^53^ Mutual synchronization not only enhances key spectral properties—such as reducing linewidth and increasing output power^2,5,54^ —but also lowers phase noise,^55^ improving the stability and scalability of these systems. Recent studies have also demonstrated the generation and control of propagating spin waves in these devices,^56,57^ underscoring their potential for use in reconfigurable magnonic conduits and spin-wave-based Ising machines.^4,58^ Additionally, advancements such as the development of ultrasmall SHNOs^37^ and the synchronization of over 100,000 oscillators in a two-dimensional array,^59^ achieving a giant quality factor and high output power (>9 nW), further highlight the miniaturization and scalability of this technology for practical applications. The highly efficient WSMs with strong SOTs could further enhance mutual synchronization while minimizing unwanted effects from HMs in bilayer SHNOs, paving the way for more efficient and scalable spintronic devices.

Moreover, due to their active topological properties, these materials can exhibit significant electric field tunability, allowing for substantial on-demand frequency adjustments using electric field gating.^10,11,60,61^ This tunability can be directly utilized to control mutual synchronization in large chains and arrays of SHNOs with wide frequency operation.^6^ The demonstrated results have implications far beyond SHNOs. The giant SOT in a single magnetic WSM with room temperature ferromagnetism is also highly useful for spintronic THz sources^16,35,62^ and three-terminal memory devices,^63^ replacing complex FM/HM heterostructures and enabling simpler, more energy-efficient designs. Overall, our demonstration will enable the use of magnetic Weyl semimetals for the further development of energy-efficient SHNOs and open new opportunities for researchers in other areas of spintronics.

## Methods

### Sample Fabrication

Epitaxial thin films of CMG (*t* = 10, 20, and 30 nm) were grown on 0.5 mm thick single-crystalline MgO (001) substrates using ultrahigh vacuum magnetron sputtering with a base pressure of less than 2 × 10^–9^ Torr. A Co–Mn–Ga alloy sputtering target was used to deposition the CMG films. The argon gas pressure during deposition was 0.1 Pa, and the deposition rate was 0.065 nm/s. All films were deposited at room temperature and subsequently annealed at 550 °C for 30 min under ultrahigh vacuum conditions. As part of the optimization process, we investigated the dependence of annealing temperature on film quality over a range of 450–650 °C and confirmed that annealing above 550 °C is necessary to achieve the *L*2_1_-ordered state. Further, to maintain a high degree of flatness in the films, we selected 550 °C as the postannealing temperature. The thickness of 10–30 nm was decided from the previous study that investigated the thickness dependence of the anomalous Hall effect.^64^ After cooling down the samples to room temperature, a 2 nm thick Al capping layer is deposited to protect the films from oxidation and damages during fabrication. *E*-Beam lithography, Ar–ion milling, and a negative e-beam resist (maN 2401) as an etching mask were then used to fabricate 500 nm wide cross Hall bars, 4 × 14 μm^2^ ST-FMR microstrips, and 150 nm wide nanoconstriction SHNOs. The Ar–ion etching is performed at a very slow rate of <0.5 Å/s to minimize damage from high-energy ions. Subsequently, the residual resist is removed using O_2_—plasma cleaning. Optical lithography is used to define the top coplanar waveguide contacts for the electrical measurements, followed by a lift-off process of 780 nm of copper and 20 nm of platinum.

### Characterization of Co_2_MnGa (CMG) Films

The composition of the CMG films is determined using X-ray fluorescence spectroscopy. The structural analysis is done using X-ray diffraction (XRD) measurements for different CMG atomic planes using different tilt angles χ. The microstructure of CMG films was analyzed using FEI Titan G2 80–200 and Thermo Fisher Scientific Spectra Ultra scanning transmission electron microscopes (STEM), operating at accelerating voltages of 200 kV and 300 kV, respectively. The longitudinal and anomalous Hall resistivities are measured using a physical property measurement system (PPMS; Quantum Design) at temperatures 50–300 K. Magnetization measurements are done using a vibrating sample magnetometer at room temperature. Broadband ferromagnetic resonance (FMR) measurements are done using a NanOsc PhaseFMR-40 system with a coplanar waveguide for broadband microwave field excitation at room temperature. Microwave excitation fields *h*_rf_ with frequencies up to 38 GHz are applied in the film plane and perpendicular to the applied in-plane dc magnetic field *H*_a_.

### Harmonic Hall Measurements

The effective fields of field-like (*H*_FL_) and antidamping-like (*H*_AD_) SOTs are evaluated using extended harmonic Hall measurements, excluding the thermoelectric effects originating due to the anomalous Nernst and spin Seebeck effects.^41,42^ The schematic of the harmonic Hall measurement setup is shown in Figure 3a, where a 213 Hz alternating current (*I*_AC_) is applied to the channel in the presence of a fixed magnetic field, μ_0_ *H*_a_. The first and second harmonic Hall voltages (*V*_ω_ and *V*_2ω_) are measured at room temperature using a lock-in-amplifier while sweeping the in-plane angle, ϕ_H_, between the *I*_AC_ and μ_0_ *H*_a_, as shown in Figure 3a.

### Magnetoresistance and Spin Torque Ferromagnetic Resonance Measurements

In-plane angular dependent anisotropic magnetoresistance measurements are performed on 4 × 14 μm^2^ ST-FMR microstrips at room temperature using a rotatable projected vector field magnet with a magnetic field magnitude of 0.1 T and an applied dc current of 0.5 mA. Room-temperature ST-FMR measurements are performed by injecting a radio frequency (rf) current to the microstrip through a high-frequency bias-*T* at a fixed frequency (ranging from 7 to 17 GHz) with an input power of *P* = 4 dBm. The rf current generates antidamping-like and field-like torques in the presence of an applied magnetic field μ_0_ *H*_a_, and the resultant torques excite the magnetization procession of the CMG film, which leads to a time-dependent change in the device resistance due to the magnetoresistance of the CMG.^47,48^ The oscillating resistance of the device mixes with the rf current and results in a dc mixing voltage, *V*_mix_, which is then measured using a lock-in-amplifier. ST-FMR measurements are performed with a fixed in-plane angle, ϕ_H_ = 60°, between the applied magnetic field and input rf/dc current.

### Micro-Brillouin Light Scattering Measurement

Magneto-optical measurements of the SHNOs are carried out using microfocused Brillouin light scattering microscopy. A monochromatic continuous wave (CW) laser (wavelength = 532 nm; laser power = 1.5 mW) was focused on the center of the nanoconstriction by a ×100 microscope objective with a large numerical aperture (NA = 0.75) down to a 300 nm diffraction limited spot diameter. The magnetic field condition was set at an in-plane angle (IP) of 0°, and an out-of-plane angle of 60°. The scattered light was analyzed with a Sandercock-type six-pass Tandem Fabry–Perot interferometer TFP-1 (from JRS Scientific Instruments). The resulting BLS intensity is proportional to the square of the amplitude of the dynamic magnetization at the location of the laser spot. A special stabilization protocol based on an active feedback algorithm (THATec Innovation) was employed to get long-term spatial stability during the μ-BLS measurements. All the measurements were performed at room temperature.

### Theoretical Calculations of Anomalous Hall and Spin Hall Conductivities

The anomalous Hall and spin Hall conductivities of CMG are calculated by combining first-principles calculations and the Kubo formula. First, we calculated the electronic structure of *L*2_1_-ordered CMG (Figure 1a) based on the density-functional theory, including the spin-orbit interaction, which is implemented in the Vienna *ab initio* simulation program (VASP).^65^ The lattice constant is set to a typical experimental value of 5.755 Å.^20^ We adopted the generalized gradient approximation for the exchange-correlation energy and used the projected augmented wave pseudopotential to treat the effect of core electrons properly. A cutoff energy of 337 eV is employed, and the Brillouin-zone integration is performed with 91 × 91 × 91 *k* points. The convergence criteria for energy and force are set to 10^–5^ eV and 10^–4^ eV/Å, respectively. Using the obtained electronic structure, we calculated the anomalous Hall and spin Hall conductivities using the following expressions derived from the Kubo formula

$$\sigma_{xy}\left(\epsilon \right)=-\frac{e^{2}}{\hbar }\int \frac{d^{3}k}{{\left(2\pi \right)}^{3}}\Omega_{xy}^{c}\left(k\right)$$ 4

5 $$\sigma_{s,xy}\left(\epsilon \right)=-\frac{e}{\hbar }\int \frac{d^{3}k}{{\left(2\pi \right)}^{3}}\Omega_{xy}^{s}\left(k\right)$$

$$\Omega_{xy}^{\alpha }\left(k\right)=-\frac{\hbar^{2}}{m^{2}}\underset{n}{\sum }f\left(E_{n,k},\epsilon \right)\underset{n^{\prime }\neq n}{\sum }\frac{2\;Im\left\langle \psi_{n,k}\left|p_{x}\right|\psi_{n^{\prime },k}\right\rangle \left\langle \psi_{n^{\prime },k}\left|p_{y}^{\alpha }\right|\psi_{n,k}\right\rangle }{{\left(E_{n^{\prime },k}-E_{n,k}\right)}^{2}}$$ 6

where σ_*xy*_(ϵ) and σ_s,*xy*_(ϵ) are the anomalous Hall and spin Hall conductivities, respectively, as a function of ϵ being the energy relative to the Fermi energy. These conductivities are given by integrating the charge Berry curvature Ω_*xy*_^c^ and the spin Berry curvature Ω_*xy*_^s^, where the generalized momentum operator *p*_*y*_^α^ is defined as *p*_*y*_^c^ = *p*_*y*_ and *p*_*y*_^s^ = {*p*_*y*_, *s*_*z*_} with the spin operator *s*_*z*_ = σ_*z*_/2.^66^ In eq 6, |ψ_*n*,**k**_⟩ is the eigenstate with the eigenenergy *E*_*n*,**k**_ for the band *n* and the wave vector **k**, and *f*(*E*_*n*,**k**_, ϵ) is the Fermi distribution function. In our calculations, the direction of the magnetization is fixed to the [001] direction, consistent with the experimental setup.

## Supplementary Material



## Data Availability

The data sets generated and/or analyzed during the current study are available from the corresponding author on reasonable request.

## Notes

Growth of epitaxial *L*2_1_ ordered Co_2_MnGa thin films; in-plane magnetization measurements at room temperature; effective magnetization using broadband ferromagnetic resonance measurements; temperature dependence of the electrical resistivity; second harmonic Hall measurements for the 20 nm CMG film; anisotropic magnetoresistance measurements; effective magnetization and Gilbert damping constant using spin-torque ferromagnetic resonance measurements; the integrated BLS counts and linewidth for 30 nm Co_2_MnGa based SHNO (PDF)
