# Supplementary material for: Energy-Efficient Single Layer Spin Hall Nano-Oscillators Driven by Berry Curvature
Source: ACS Nano. 2025 May 9;19(19):18534–44. doi: 10.1021/acsnano.5c02048 (PMC12096435; doi:10.1021/acsnano.5c02048)
Supplement: Supplementary file 1 [file nn5c02048_si_001.pdf]

Supporting Information for

Energy-efficient single layer spin Hall nano-oscillators driven by Berry curvature

Lakhan Bainsla,[*,†,‡,¶,△] Yuya Sakuraba,[*,§,△] Akash Kumar,[‡,∥,⊥,△] Avinash Kumar Chaurasiya,[‡,△] Keisuke Masuda,[§] Nattamon Suwannaharn,[§] Ahmad A. Awad,[‡,∥,⊥] Nilamani Behera,[‡] Roman Khymyn,[‡] Taisuke Sasaki,[§] Saroj Prasad Dash,[¶,#,@] and Johan Åkerman[*,‡,∥,⊥]

[†]Department of Physics, Indian Institute of Technology, Ropar, Roopnagar, 140001, India
[‡] Department of Physics, University of Gothenburg, Göteborg, 41296, Sweden
[¶]Department of Microtechnology and Nanoscience, Chalmers University of Technology, Göteborg, 41296, Sweden
[§]Research Center for Magnetic and Spintronic Materials, National Institute for Materials Science, 1-2-1, Sengen, Tsukuba, 305-0047, Ibaraki, Japan
[∥]Center for Science and Innovation in Spintronics, Tohoku University, 2-1-1 Katahira, Aoba-ku, Sendai, 980-8577, Japan
[⊥]Research Institute of Electrical Communication, Tohoku University, 2-1-1 Katahira, Aoba-ku, Sendai, 980-8577, Japan
[#]Wallenberg Initiative Materials Science for Sustainability, Department of Microtechnology and Nanoscience, Chalmers University of Technology, Göteborg, 41296, Sweden
[@]Graphene Center, Chalmers University of Technology, Göteborg, 41296, Sweden

[△]These authors contributed equally to this work
E-mail: lakhan.bainsla@iitrpr.ac.in; SAKURABA.Yuya@nims.go.jp; johan.akerman@physics.gu.se

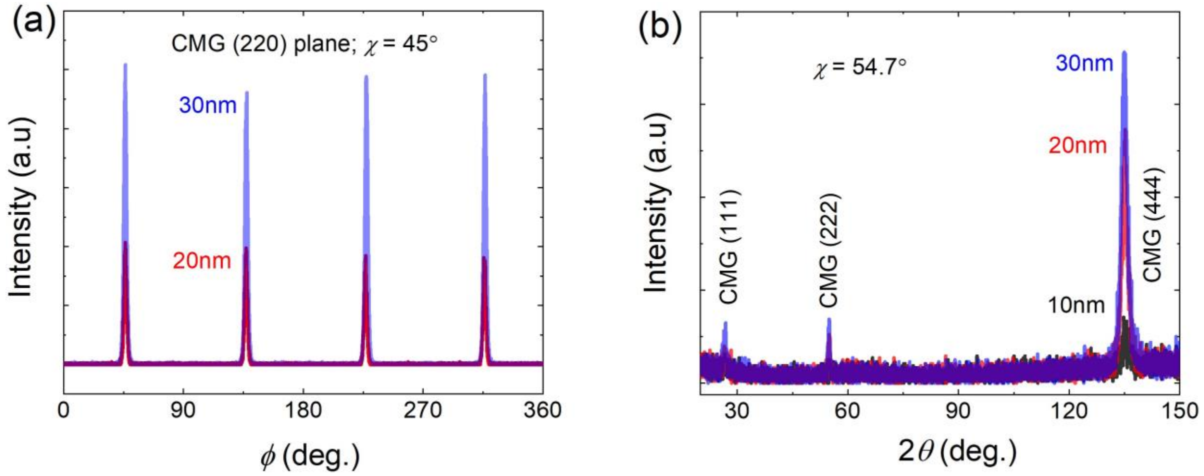

Figure S1: Growth of epitaxial $L2_1$ ordered Co_2MnGa thin films. **(a)** ϕ-scan data for the CMG (220) plane measured for the 20 and 30 nm films, respectively. **(b)** The out-of-plane XRD patterns for the CMG (111) plane measured for the 10, 20, and 30 nm films, respectively.

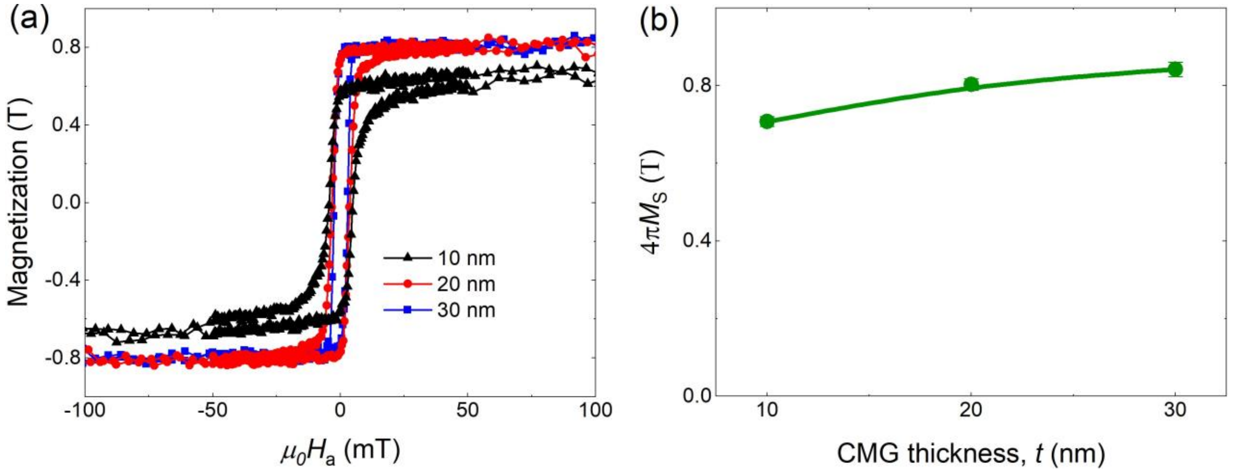

Figure S2: In-plane magnetization measurements at room temperature. **(a)** In- plane magnetization at 300 K for the different CMG thicknesses. **(b)** Saturation magnetization*vs.* film thickness; lines are guides to the eye.

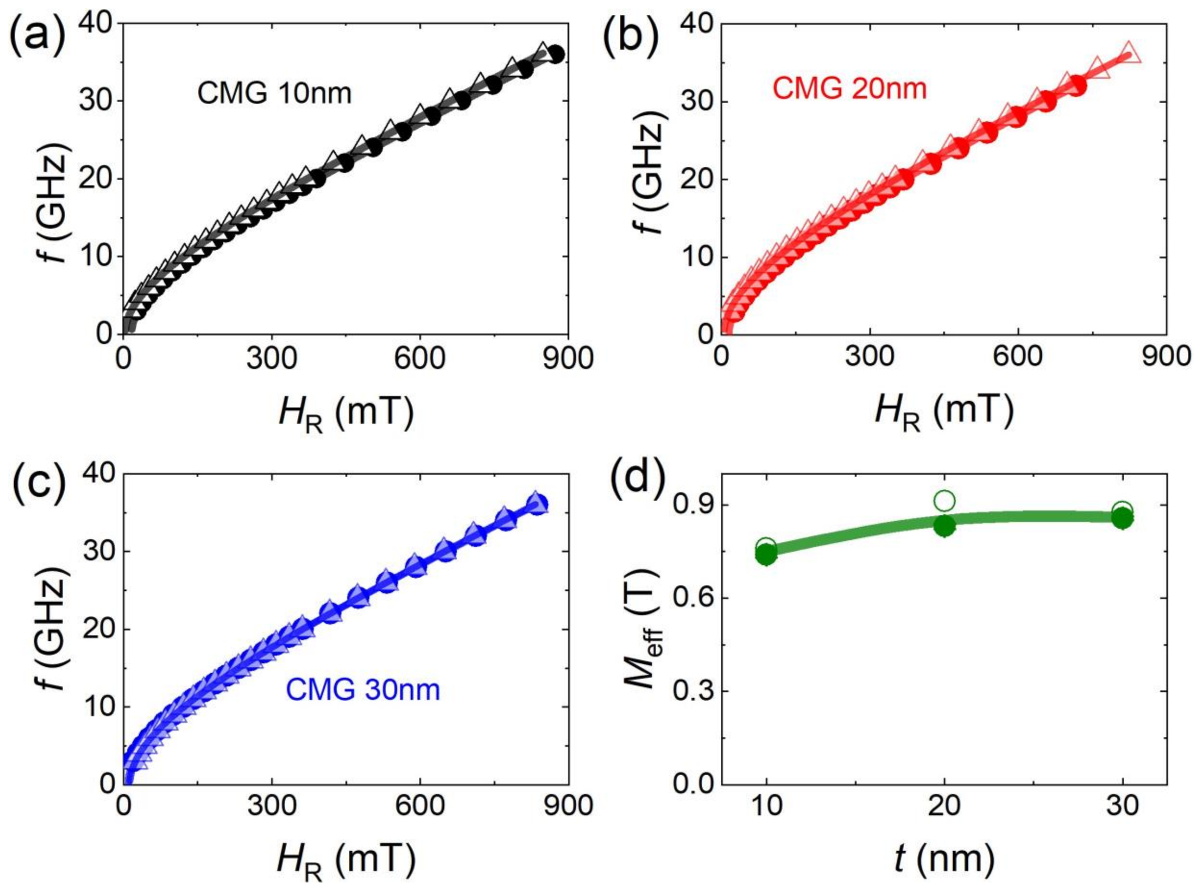

Figure S3: Effective magnetization using broadband ferromagnetic resonance measurements. **(a-c)**, Frequency, *f*, *vs.* ferromagnetic resonance field, H_R for the 10, 20, and 30 nm films, respectively. **(d)** Extracted values of effective magnetization, $\mu_0 M_{eff}$, *vs.* CMG thickness *t*. **(a-d)** Solid and open symbols represent the experimental data points when appliedmagnetic field, $\mu_0 H_a$, is parallel to CMG (110) and (001) planes. **(a-c)** solid lines are fits toKittel's equation; while solid line in **(d)** just a guide to eye.

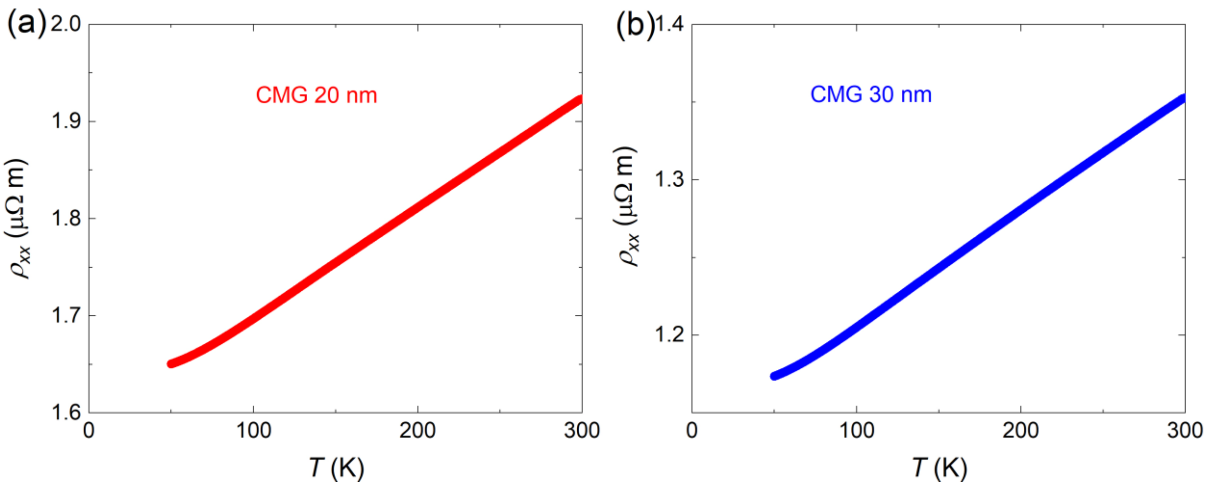

Figure S4: Temperature dependence of the electrical resistivity. **(a-b)** Temper- ature dependence of electrical resistivity for the 20 and 30 nm Co_2MnGa films, respectively, inthe temperature range of 50-300 K.

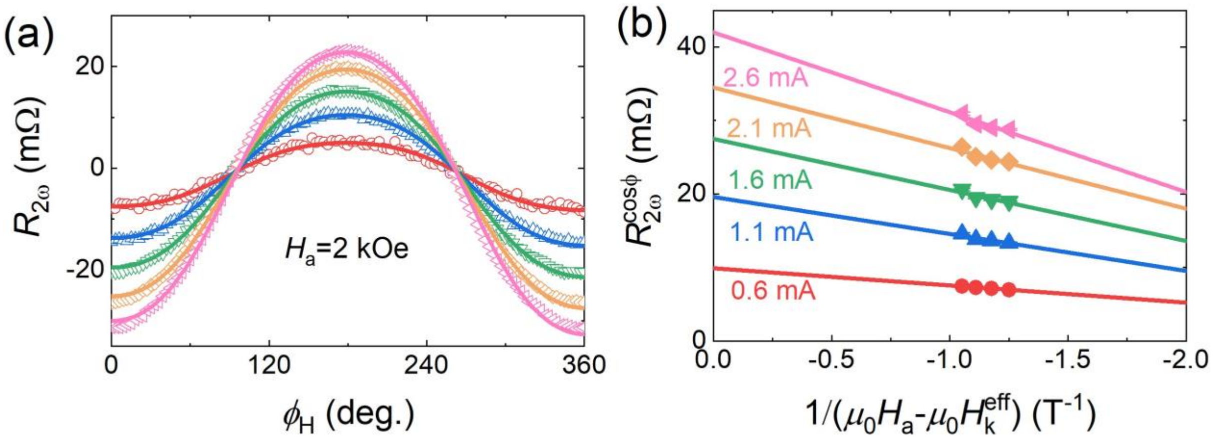

Figure S5: Second harmonic Hall measurements for the 20-nm CMG film. **(a)**Second harmonic hall resistance, $R_{2\omega}$, of the 20-nm CMG film *vs.* ϕ_H with $\mu_0 H_a$ = 0.2 T, for five different alternating current values I_{AC} = 0.6-2.6 m*A*, in steps of 0.6 m*A*. Solid symbols are the experimental data points and solid lines are fit to Eq. 1. **(b)** The $\cos\phi$ contribution to $R_{2\omega}$ ($R_{2\omega}^{\cos\phi}$) as a function of $1/(\mu_0 H_a - \mu_0 H^{eff})$, here filled symbols are the experimental data points; solid lines are linear fits.

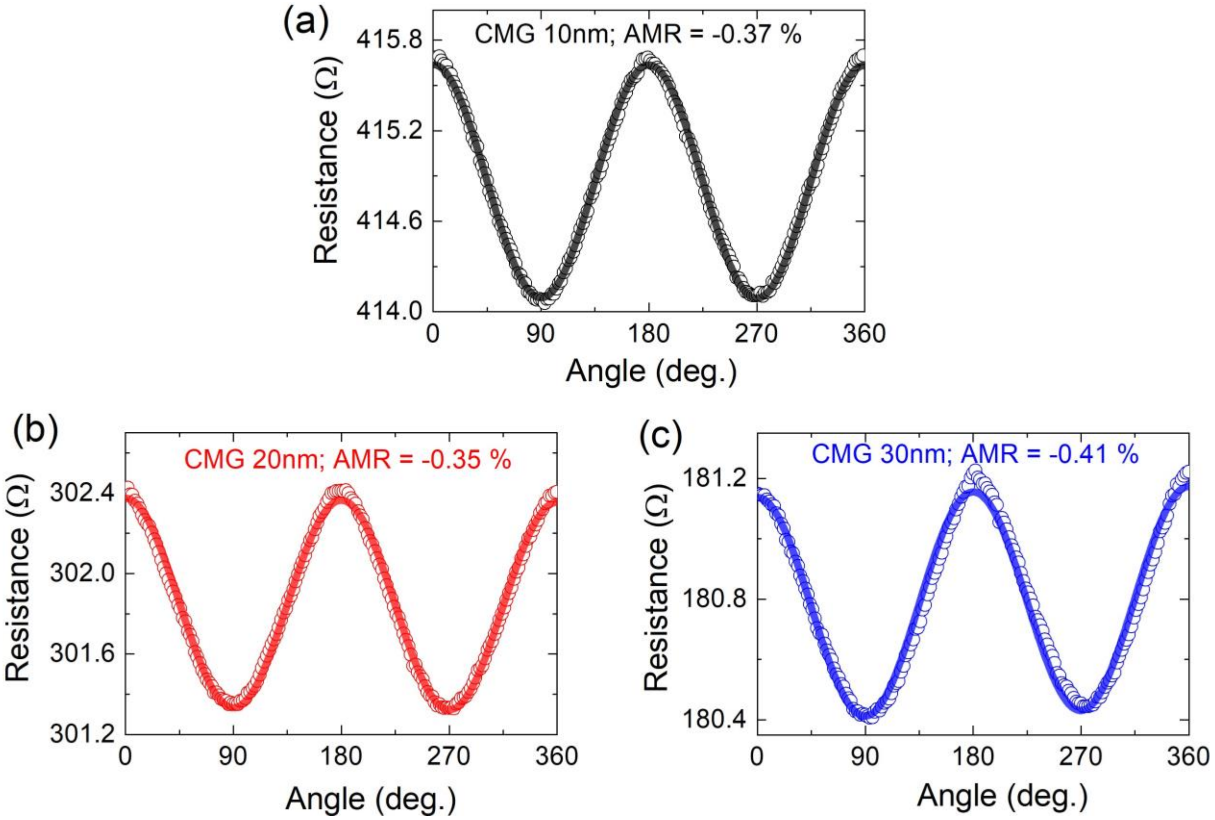

Figure S6: Anisotropic magnetoresistance measurements. **(a-c)** The resistanceof the ST-FMR micro bars as a function of in-plane angle, ϕ_H for CMG 10, 20, and 30 nm films, respectively. The solid symbols represent the experimental data and the solid lines are sinusoidal fits to the data. The measurements are done with an applied magnetic field of 100 mT and dc current of 0.5 mA.

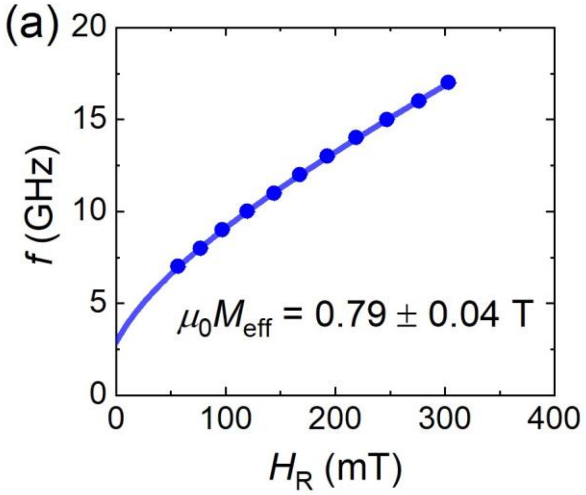

Figure S7: Effective magnetization and Gilbert damping constant using spin- torque ferromagnetic resonance measurements. **(a-b)** Frequency f *vs.* resonance field H_R and resonance linewidth ΔH *vs.* frequency f, respectively. Here, the solid symbols show the values obtained by fitting the V_{mix} signal, and the solid lines show the fit to the data. The obtained values of effective magnetization, $\mu_0 M_\mathrm{eff}$ and the Gilbert damping constant α are shown in Fig. 7a and 7b, respectively. All the measurements are done with $\phi_\mathrm{H} = 60^\circ$.

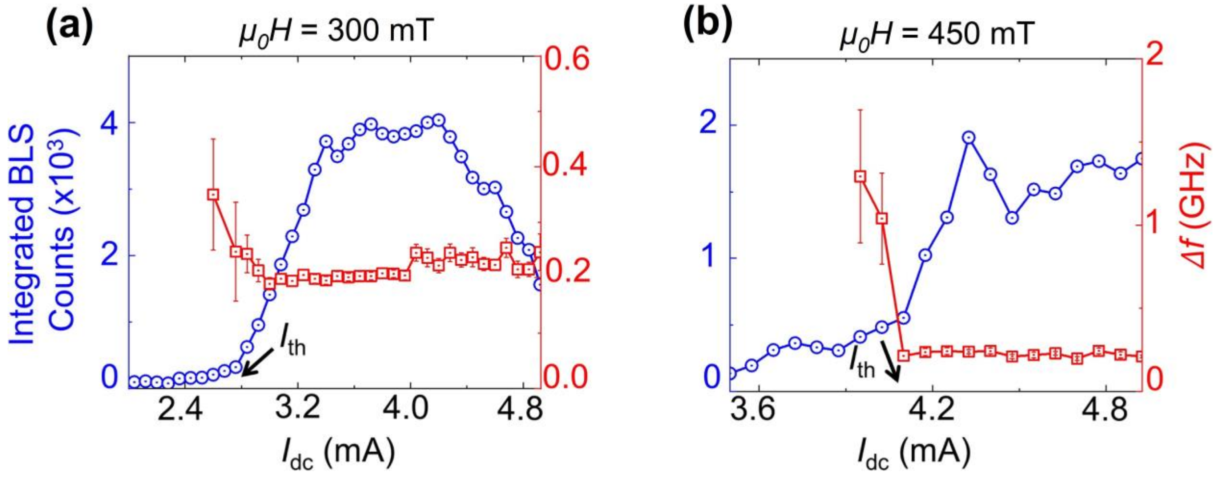

Figure S8: The integrated BLS counts and linewidth for 30 nm Co_2MnGa basedSHNO. **(a-b)** The integrated BLS counts and linewidth as a function of I_{dc} for $\mu_0 H$ = 300 mT, and 450 mT, respectively.
